# A Survey of Availability and Affordability of Polypills for Cardiovascular Disease in Selected Countries

**DOI:** 10.5334/gh.1335

**Published:** 2024-07-01

**Authors:** Gautam Satheesh, Bishal Gyawali, Marie France Chan Sun, Mark D. Huffman, Amitava Banerjee, Pablo Perel, Adrianna Murphy

**Affiliations:** 1 The George Institute for Global Health, Hyderabad, India; 2 Global Health Section, Department of Public Health, University of Copenhagen, Copenhagen, Denmark; 3 Department of Medicine, University of Mauritius, Mauritius; 4 Washington University in St. Louis, St. Louis, Missouri, USA; 5 The George Institute for Global Health, University of New South Wales, Sydney, New South Wales, Australia; 6 Institute of Health Informatics, University College London, London, UK; 7 Department of Non-Communicable Disease Epidemiology, London School of Hygiene and Tropical Medicine, London, UK; 8 World Heart Federation, Geneva, Switzerland; 9 Department of Health Service Research and Policy, Faculty of Public Health and Policy, London School of Hygiene and Tropical Medicine, London, UK

**Keywords:** Polypill, Cardiovascular disease, Essential Medicines, Access, Secondary Prevention

## Abstract

**Background:**

The recent inclusion of polypills—fixed-dose combinations of antihypertensive medicines and a statin with or without aspirin—in the World Health Organization’s Essential Medicines List (EML) reiterates the potential of this approach to improve global treatment coverage for cardiovascular diseases (CVDs). Although there exists extensive evidence on the effectiveness, safety and acceptability of polypills, there has been no research to date assessing the real-world availability and affordability of polypills globally.

**Methods:**

We conducted a cross-sectional survey, based on the WHO/Health Action International methodology, in 13 countries around the world. In the surveyed countries, we first ascertained whether any polypill was authorised for marketing and/or included in EMLs and clinical guidelines. In each country, we collected retail and price data for polypills from at least one public-sector facility and three private pharmacies using convenience sampling. Polypills were considered unaffordable if the lowest-paid worker spent more than a day’s wage to purchase a monthly supply.

**Results:**

Polypills were approved for marketing in four of the 13 surveyed countries: Spain, India, Mauritius and Argentina. None of these countries included polypills in national guidelines, formularies, or EMLs. In the four countries, no surveyed public pharmacies stocked polypills. In the private sector, we identified seven unique polypill combinations, marketed by eight different companies. Private sector availability was 100% in Argentina and Spain. Most combinations (n = 5) identified were in India. Combinations found in India and Spain were affordable in the local context. A lowest-paid government worker would spend between 0.2 (India) and 2.8 (Mauritius) days’ wages to pay the price for one month’s supply of the polypills. Polypills were likely to be affordable if they were manufactured in the same country.

**Conclusion:**

Low availability and affordability of polypills in the public sector suggest that implementation remains poor globally. Context-specific multi-disciplinary health system research is required to understand factors affecting polypill implementation and to design and evaluate appropriate implementation strategies.

## Introduction

Cardiovascular disease (CVD) is the leading cause of death worldwide and a major contributor to poor health and disability [1,2]. In low- and middle-income countries (LMICs), premature mortality from CVD is on the rise. This increase is partly due to persistent gaps in treatment; in LMICs, less than one third of the individuals who are eligible for drug therapy for primary and secondary prevention of CVD are actually taking the recommended medicines [3]. Poor access to healthcare, including risk screening and diagnosis of CVD, is one important cause of gaps in treatment. However, for those who do access care, the number of medicines required for effective treatment, the high costs of treatment, and variations in health worker capacity and prescribing practices can all act as barriers to treatment prescription and adherence [4]. While overall death rates from CVD have declined in the last two decades in high-income countries because of improved use of appropriate treatment, treatment coverage remains suboptimal globally [5].

The cardiovascular polypill, defined as a fixed-dose combination of antihypertensive medicines and a statin, with or without aspirin, offers one potential tool to address barriers to treatment adherence. The concept of combining antihypertensives with statins and aspirin for the secondary prevention of CVD was first introduced by the World Health Organization (WHO) in 2001 [6] as a means of improving treatment consistency and adherence. The idea was further developed by Wald and Law in 2003 [7], when they proposed the polypill as a population-level strategy for primary prevention of CVD. Since then, various randomised controlled trials have demonstrated the safety and effectiveness of the polypill among those with or at risk of atherosclerotic CVD, including significant reductions in all-cause mortality, major adverse cardiovascular events, blood pressure, and blood cholesterol and improved medication adherence [8,9]. The 2023 European Society of Cardiology guidelines for the management of acute coronary syndromes emphasise the role of polypills in improving adherence [10]. Polypills also have the potential to be cost-effective. In a UK study, implementation of the polypill for primary prevention of CVD at the cost of USD 1.30 per person was associated with a net saving of USD 2500 per year of life saved by preventing a first myocardial infarction or stroke [11]. In light of evidence supporting the use of the polypill, three combinations for prevention of atherosclerotic CVD have been added to the 2023 WHO Model Essential Medicine List [12].

Despite supporting evidence, however, implementation of the polypill globally is poor. Although there are now several polypills that have been developed and studied in clinical trials [8,9] and evidence of the acceptability of this approach among patients and prescribers [13], there has been no research to-date assessing the real-world availability and affordability of polypills around the world (i.e., availability outside of research study settings). Insight into these areas is needed to inform discussions of the feasibility of recommending polypill use and to better understand where barriers to implementation may lie. We aimed to document the availability and affordability of polypills in selected countries.

## Methods

### Investigator team

This study was led by members of the World Heart Federation Salim Yusuf Emerging Leaders Programme. The programme is a network of researchers and practitioners from around the world who have demonstrated a commitment to reducing CVD mortality and morbidity.

### Survey setting

The survey was conducted in 13 countries between June and September 2022. Emerging Leaders are based in over 50 countries, and all were invited to participate. The 13 countries included were selected based on where the Emerging Leaders who were willing and available to contribute to the study were based, aiming as much as possible to get representation from every region of the world, namely, Bangladesh, Kenya, India, Nepal, Iraq, Nigeria, Cameroon, Mauritius, Sweden, Spain, Argentina, Colombia and Mexico.

### Survey design

A cross-sectional survey was developed based on the WHO/Health Action International (WHO/HAI) methodology and adapted for feasibility [14,15]. In each selected country, 1–2 Emerging Leaders used a standardised survey tool to capture data on market authorization status, availability, brands, prices, affordability and adoption (in guidelines and national EMLs) of the polypill. All Emerging Leaders received training on the study methods and the survey tool, and the survey tool was piloted in a small sample of private-sector facilities in India and Nepal. For this study, the polypill was defined as a fixed-dose combination containing two or more antihypertensive medicines and a statin, with or without aspirin. Recognising that polypills of that definition were unlikely to be commonly available, we also collected data on two-pill combinations of one antihypertensive and one cholesterol-lowering medicine.

The survey included an initial filtering question for the Emerging Leaders to ascertain whether any polypills or two-pill combinations that fit our study definition were authorised for marketing in the country. Further survey questions covered inclusion in essential medicines lists and clinical guidelines, retail availability and the price of polypills. Retail and price data were collected from at least one public sector facility (preferably a large tertiary care hospital in the region by bed strength) and at least three private pharmacies using convenience sampling. Where data collection from tertiary care hospitals was not possible, data were collected from other public sector facilities. Price data were obtained from facilities even if the polypills or two-pill combinations were out of stock.

### Data analysis

Data were analysed by country to provide descriptive estimates of polypill availability and affordability. Availability was defined as the number of facilities in a given sector that stocked any polypill on the day of the survey. Affordability was defined using the WHO/HAI standards, according to which a medicine is affordable if the cost of one month’s supply is lower than the lowest daily wage of a government worker in that state or country. The cost of one month’s supply of polypills was calculated by assuming a single daily dose. The complete survey tool, including instructions for data collectors, is available as a supplementary file.

### Ethical considerations

The study did not require an ethical review as it was not considered human subjects research and no human data were collected. Emerging Leaders explained the purpose of the study to the facility managers and obtained verbal consent to collect medicine data, which are publicly available.

## Results

Polypills were approved for marketing in four of the 13 surveyed countries: Spain, India, Mauritius and Argentina. However, none of these countries included polypills in their national guidelines, formularies or EMLs.

### Availability

None of the surveyed public pharmacies in any of the four countries stocked any polypills for primary or secondary prevention of CVD; however, polypills were stocked in the private pharmacies in each of these countries. Private sector availability ranged from 0% in India (out-of-stock on the survey day but listed for sale) to 100% in Argentina and Spain. The types of polypills available in private pharmacies are shown in Table 1. Overall, we identified seven unique polypill combinations in the four countries where polypills were authorised, marketed by eight different companies. Most combinations (n = 5) identified were in India.

**Table 1:** Availability and affordability of polypills stocked in private pharmacies in Spain, India, Mauritius, and Argentina.


TYPE	AVAILABILITY (%) INDICATION		MANUFACTURING COUNTRY AND COMPANY	AFFORDABILITY			
	
	*PUBLIC SECTOR*	*PRIVATE SECTOR*		MEDIAN PRICE/TAB (USD)	COST FOR 1 MONTH SUPPLY (USD)	MINIMUM DAILY WAGE (USD)	NUMBER OF DAYS’ WAGES

***Argentina***	***N = 1***	***N = 3***					

Rosuvastatin + Candesartan + Hydrochlorothiazide	0.0	100.0	Argentina (Lepetit)	0.31	9.30	7.64	1.21

***India***	***N = 5***	***N = 5***								

Aspirin + Atorvastatin + Ramipril	0.0	0.0*	India (Zydus Lifesciences)	0.04	1.26	5.41	0.23
	
Aspirin + Simvastatin + Ramipril + Atenolol + Hydrochlorothiazide	0.0	0.0*	India (Cadila)	0.34	10.14		1.87
	
Atorvastatin + Ramipril + Metoprolol	0.0	0.0*	India (Zydus Lifesciences, Emcure)	0.13	4.04		0.75
	
Aspirin + Atorvastatin + Ramipril + Metoprolol	0.0	0.0*	India (Torrent, Zydus Lifesciences)	0.12	3.60		0.67
	
Aspirin + Atorvastatin + Losartan	0.0	0.0*	India (Cipla)	0.17	5.22		0.96

***Mauritius***	***N = 5***	***N = 15***					

Atorvastatin + Perindopril + Amlodipine	0.0	13.33	France (Servier)	0.88	26.46	9.52	2.78

***Spain***	***N = 1***	***N = 8***					

Aspirin + Atorvastatin + Ramipril	0.0	100.0	Spain (Ferrer)	0.74	22.20	32.66	0.68


Polypills whose monthly supply cost more than one day’s wage of the lowest-paid government worker are provided in red. *The medicine was physically out-of-stock on the survey day, but the pharmacist was able to provide price data as the medicine is usually stocked.

### Affordability

The prices and affordability of available polypills are also shown in Table 1. A lowest-paid government worker would spend between 0.2 (India) and 2.8 (Mauritius) days’ wages to pay the price for a month’s supply of the polypill. Many combinations (63%) found in this survey were affordable in the local context, but these were limited to India and Spain. Polypills that were locally unaffordable were found in Mauritius, Argentina (1.2 days’ wages) and India (1.9 days’ wages). Polypills were likely to be affordable within a given country if they were manufactured in the same country.

### Two-pill combinations

Among the surveyed countries, two-pill combinations were approved for marketing in Argentina, Bangladesh, Cameroon, India, Mauritius and Spain but were not included in national guidelines, essential medicine lists or in public pharmacies in these or any other countries in our study. The two-pill combinations were stocked by private pharmacies in Argentina, Bangladesh, Cameroon, India and Spain. They were also stocked in private pharmacies in Nepal but without marketing authorisation. We identified eight unique combinations in total, all of which were identified in India (Table 2). Costs ranged from less than 1.0 day’s wages in India to 7.1 days in Cameroon. A total of 64% of two-pill combinations were affordable in the local context, but these were limited to India, Nepal and Spain.

**Table 2:** Availability and affordability of two-pill combinations stocked in private pharmacies in six countries.


TYPE	PUBLIC SECTOR AVAILABILITY (%)	PRIVATE SECTOR AVAILABILITY (%)	MANUFACTURING COMPANIES	MEDIAN PRICE/TAB (USD)	COST OF 1 MONTH SUPPLY	LOWEST WAGE (USD)	NUMBER OF DAYS’ WAGES

***Argentina***	***N = 1***	***N = 3***					

Atorvastatin + Amlodipine	0.0	100.0	Casaco laboratorios	1.00	30.00	7.64	3.93

***Bangladesh***	***N = 3***	***N = 5***					

Atorvastatin + Amlodipine	0.0	71.4	Delta Pharma, Beximco	0.073	2.19	0.46	4.76

***Cameroon***	***N = 8***	***N = 10***					

Atorvastatin + Amlodipine	0.0	20.0	Pfizer, Ajanta Pharma Limited	0.41	12.45	1.75	7.11

***India***	***N = 5***	***N = 5***					

Atorvastatin + Amlodipine	0.0	40.0	Torrent, Alembic	0.11	3.35	5.41	0.62
	
Atorvastatin + Atenolol	0.0	0.0*	Numerous brands	0.08	2.40		0.44
	
Atorvastatin + Losartan	0.0	0.0*	Numerous brands	0.04	1.33		0.25
	
Atorvastatin + Metoprolol	0.0	0.0*	Numerous brands	0.11	3.16		0.58
	
Atorvastatin + Olmesartan	0.0	0.0*	Numerous brands	0.11	3.45		0.64
	
Atorvastatin + Ramipril	0.0	20.0	Sanofi India	0.32	9.54		1.76
	
Atorvastatin +Telmisartan	0.0	0.0*	Numerous brands	0.12	3.56		0.66
	
Rosuvastatin +Telmisartan	0.0	0.0*	Numerous brands	0.18	5.40		1.00

***Nepal***	***N = 5***	***N = 5***					

Atorvastatin + Amlodipine	0.0	0.0*	Numerous brands	0.12	3.51	4.42	0.79

***Spain***	***N = 1***	***N = 8***					

Atorvastatin + Amlodipine	0.0	100.0	Viatris Healthcare; Almirall S.A; Krka, d. d., Novo mesto	0.50	15	32.85	0.46

Rosuvastatin + Amlodipine	0.0	100.0	Teva Pharmaceuticals	0.50	15	32.85	0.46


*The medicine was physically out-of-stock on the survey day, but the pharmacist was able to provide price data as the medicine is usually stocked.

## Discussion

Our study used practical methods to assess the availability and affordability of polypills in selected countries from almost all WHO regions. Of the 13 countries included in our survey, we identified only four countries where polypills are approved for marketing. In these countries, they were available only in the private sector. While several polypills found in sampled facilities were unaffordable in the local context, there are a range of affordable polypills found in India and one in Spain. These findings highlight poor overall uptake of polypills and the importance of taking a health system-wide perspective when developing strategies to improve access to polypills.

Market failure is one major cause of the poor availability of polypills [16,17,18]. The potential profit margin for companies to invest in the development of polypills is low, as its individual components are generics, which have higher competition and relatively lower prices. Low market incentives limit market entry. Entry into foreign markets may also be impeded by cross-country variability in regulatory frameworks for drug approval, requirements for clinical trials and safety and effectiveness data, and intellectual property, trade, and pricing laws. Some countries simply do not provide regulatory approval for any fixed dose combinations, while others lack the resources or efforts of the national regulatory agencies. In our survey, authorisation and market availability were indeed more likely in the countries where manufacturers of the polypill are based. However, we know that polypills are marketed in many countries not included in this survey and where they are not produced locally. For example, the formulation of acetylsalicylic acid, atorvastatin and ramipril manufactured by Ferrer Internacional, S.A. of Spain, is authorised for marketing for secondary prevention under the names Trinomia, Sincronium and Iltria in 26 countries worldwide, including Spain and Mexico, that we surveyed [16].^1^ An additional proposed formulation is the fixed-dose combination of atorvastatin, perindopril and amlodipine, manufactured by Servier Laboratories, France, and authorised for marketing for primary prevention under the names Triveram and Lipertance in 51 countries [12].^2^

Importantly, though, we find that even where polypills are authorised for marketing, this does not translate into availability. Our study found no polypills available in public outlets in countries where they were authorised. It has been reported that of the 26 countries where Ferrer’s polypill has market authorisation, only one (Mexico) uses the polypill in the public sector [16]. The experiences of improving access to other generic essential medicines have shown that implementation of medicines in the public sector is a complex problem, involving stakeholders at all levels of the health system [18].

Some health system interventions that are essential for promoting polypill implementation are known. At the global level, inclusion in the WHO Model EML has been a crucial step in triggering the adoption of medicines in national health care systems [19] and may prove to be so in the case of the polypill. The inclusion of two-pill fixed dose combinations for treatment of hypertension on the WHO Model EML in 2019 showed the impact that inclusion on the WHO Model EML can have on adoption at the global level; fixed dose combinations are now recommended by all major international clinical practice guidelines for hypertension [21] and were included in the short list of medicines targeted in a regional pooled procurement initiative led by the Strategic Fund of the Pan American Health Organization [19]. Yet, as is also the case with combinations for hypertension, inclusion on the WHO EML may not lead directly to access to polypills at the national level. Polypills should also be included on national essential medicine lists and clinical guidelines, regional essential medicine lists and guidelines (where applicable), and in medical school curricula. The omission of medicines in these key policy tools delays their inclusion in local formularies, procurement and supply chains, and reimbursement systems, further exacerbating poor market viability and low demand [16,19]. Simple standardised treatment protocols for CVD that reach millions at the primary care level should be prioritised over individualised ‘specialist’ treatment, especially in resource-limited settings, and the polypills can support this approach [19,20]. Other previously proposed strategies include: (i) price benchmarking, i.e., establishing a maximum retail price for medicines, as in India and China; (ii) promoting generic market competition; (iii) streamlining the regulatory system to win stakeholders’ confidence; and (iv) establishing prescribing targets for polypills and aligning incentives among prescribers and consumers [19]. However, these are all rather slow and elaborate processes that vary distinctly across countries and require a comprehensive understanding of the mechanisms and strategies to align the motivations of key actors.

There are likely other important health system factors affecting polypill implementation that are more difficult to define or modify. These are the ‘software’ of health systems [19]—values and norms, and human relations and interactions. Some of these factors are known. Among physicians, for example, there is a perception that polypills limit autonomous clinical decision-making and flexible/individualised prescribing [22]. Addressing this requires coordinated efforts, led by national professional societies and supported by international organisations, to better inform health care professionals about the range of polypills available on the market and the evidence supporting their use for individuals at different levels of CVD risk. Physicians should also consider the adaptability of polypills within their existing clinical frameworks. Understanding other factors specific to national and local health systems requires context-specific analyses of implementation stakeholders and processes using multi-disciplinary methods and frameworks [19,23]. Critically, there will be no one-size-fits-all solution to effective implementation of polypills; multi-level strategies will have to be co-designed and tested with local stakeholders. We must also learn from what has not worked [19] and align research and advocacy efforts with the needs of those making policy and those who stand to benefit most from the polypill.

### Limitations

This study has some limitations. First, our selection of countries was not random, and data on availability and prices of polypills and two-pill combinations were collected at a specific time point and only in a single city or district in each country. As a result, our findings may have limited representativeness, as this may not reflect the longitudinal availability of the polypill. We also know that the polypill has been authorised for marketing in some countries not included in our survey. However, we were limited by the availability of data collectors, and our findings are consistent with anecdotal evidence of poor uptake of the polypill, particularly in the public sector. Second, the affordability calculation employed the lowest daily wage of a government worker, which might be an overestimate as many informal workers earn less than a government daily wage worker. As such, the polypill could be less affordable than our findings suggest. Third, while we found polypills available in some private sector facilities, our findings do not reflect the actual use and prescribing patterns in these facilities, as assessing these was beyond the scope of our study. Considering the large research gaps pertaining to polypills, especially in LMICs, future cross-sectional surveys and mixed-methods studies must focus on utilisation and prescribing patterns. Nonetheless, this is one of the first studies to report the availability of polypills in various countries.

## Conclusion

Low availability and affordability of polypills in the public sector suggest that implementation remains poor globally. Context-specific multi-disciplinary health system research is required to understand factors affecting polypill implementation and to design and evaluate appropriate implementation strategies.

## Additional File

The additional file for this article can be found as follows:

10.5334/gh.1335.s1 Supplementary file. Survey data collection tool.

## Footnotes

Mexico, Chile, Costa Rica, Dominican Republic, El Salvador, Guatemala, Honduras, Austria, Belgium, Germany, Greece, Ireland, Portugal, Serbia, Spain; Armenia, Belarus, Bosnia-Herzegovina, Georgia, Jordan, Kazakhstan, Kosovo, Moldova, Montenegro, Ukraine, and Uzbekistan.

Armenia, Belgium, Bulgaria, Cambodia, Antigua and Barbuda, Bahamas, Barbados, Cuba, Dominica, Dominican Republic, Grenada, Haiti, Jamaica, Saint Kitts and Nevis, Saint Lucia, Saint Vincent and the Grenadines, Trinidad and Tobago, Belize, Costa Rica, El Salvador, Guatemala, Honduras, Nicaragua, Panama, Croatia, Chechia, Estonia, Finland, France, Germany, Ghana, Greece, Ireland, Italy, Ivory Coast, Kazakhstan, Latvia, Lithuania, Luxembourg, Madagascar, Mauritius, Myanmar, Paraguay, Philippines, Poland, Portugal, Russia, Slovakia, Slovenia, Switzerland, Vietnam.

## References

[1] Forouzanfar M, Murray CJL, Vos T, Alexander L, Bachman V, Biryukov S, et al. Global, regional, and national comparative risk assessment of 79 behavioural, environmental and occupational, and metabolic risks or clusters of risks in 188 countries, 1990–2013: A systematic analysis for the Global Burden of Disease Study 2013. Lancet. 2015; 386(10010): 2287–2323. DOI: 10.1016/S0140-6736(15)00128-2 26364544 PMC4685753

[2] Roth GA, Johnson C, Abajobir A, Abd-Allah F, Abera SF, Abyu G, et al. Global, regional, and national burden of cardiovascular diseases for 10 causes, 1990 to 2015. Journal of the American College of Cardiology. 2017; 70(1): 1–25. DOI: 10.1016/j.jacc.2017.04.052 28527533 PMC5491406

[3] Zhu JZ, Manne-Goehler J, Agarwal A, Bahendeka SK, Damasceno A, Marcus ME, et al. Medication use for cardiovascular disease prevention in 40 low- and middle-income countries. Journal of the American College of Cardiology. 2023; 81(6): 620–622. DOI: 10.1016/j.jacc.2022.12.003 36754520 PMC9990142

[4] Banerjee A, Khandelwal S, Nambiar L, Saxena M, Peck V, Moniruzzaman M, et al. Health system barriers and facilitators to medication adherence for the secondary prevention of cardiovascular disease: A systematic review. Open Heart. 2016; 3: e000438. DOI: 10.1136/openhrt-2016-000438 27738515 PMC5030589

[5] Jagannathan R, Patel SA, Ali MK, Narayan KM. Global updates on cardiovascular disease mortality trends and attribution of traditional risk factors. Current Diabetes Reports. 2019; 19: 44. DOI: 10.1007/s11892-019-1161-2 31222515

[6] World Health Organization. Secondary prevention of non-communicable disease in low and middle income countries through community-based and health service interventions. Geneva. 2002; 1–3 August.

[7] Wald NJ, Law MR. A strategy to reduce cardiovascular disease by more than 80%. BMJ. 2003; 326(7404): 1419. DOI: 10.1136/bmj.326.7404.1419 12829553 PMC162259

[8] Rao S, Siddiqi TJ, Khan MS, Michos ED, Navar AM, Wang TJ, et al. Association of polypill therapy with cardiovascular outcomes, mortality, and adherence: A systematic review and meta-analysis of randomized controlled trials. Progress in Cardiovascular Diseases. 2022; 73: 48–55. DOI: 10.1016/j.pcad.2022.01.005 35114251 PMC10535365

[9] Joseph P, Roshandel G, Gao P, Pais P, Lonn E, Xavier D, et al. Fixed-dose combination therapies with and without aspirin for primary prevention of cardiovascular disease: An individual participant data meta-analysis. Lancet. 2021; 398(10306): 1133–1146. DOI: 10.1016/S0140-6736(21)01827-4 34469765

[10] Byrne RA, Rossello X, Coughlan JJ, Barbato E, Berry C, Chieffo A, et al. 2023 ESC Guidelines for the management of acute coronary syndromes. European Heart Journal. 2023; 44(38): 3720–3826. DOI: 10.1093/eurheartj/ehad191 37622654

[11] Wald NJ, Luteij JM, Morris JK, Taylor D, Oppenheimer P. Cost–benefit analysis of the polypill in the primary prevention of myocardial infarction and stroke. European Journal of Epidemiology. 2016; 31: 415–426. DOI: 10.1007/s10654-016-0122-1 26946426 PMC4877433

[12] Huffman M, Agarwal A, Zhu JJ, et al. An application to include fixed dose combinations in the WHO Model List of Essential Medicines for primary and secondary prevention of atherosclerotic cardiovascular diseases in adults; 2023. Available from: https://cdn.who.int/media/docs/default-source/essential-medicines/2023-eml-expert-committee/applications-for-addition-of-new-medicines/a16_cvd-fdc.pdf?sfvrsn=a9d4588_2.

[13] Murphy A, Willis R, Ansbro E, Masri S, Kabbara N, Dabbousy T, et al. Implementation of fixed-dose combination therapy for secondary prevention of atherosclerotic cardiovascular disease among Syrian refugees in Lebanon: a qualitative evaluation. BMC Health Services Research. 2022; 22(1): 744. DOI: 10.1186/s12913-022-08040-z 35659222 PMC9167520

[14] WHO/HAI. Measuring medicine prices, availability, affordability and price components. 2^nd^ ed. Geneva, Switzerland: WHO; 2008.

[15] van Mourik MS, Cameron A, Ewen M, Laing RO. Availability, price and affordability of cardiovascular medicines: a comparison across 36 countries using WHO/HAI data. BMC Cardiovascular Disorders. 2010; 10: 25. DOI: 10.1186/1471-2261-10-25 20534118 PMC2898673

[16] Webster R, Castellano JM, Onuma OK. Putting polypills into practice: Challenges and lessons learned. Lancet. 2017; 389(10073): 1066–1074. DOI: 10.1016/S0140-6736(17)30558-5 28290996

[17] Webster R, Rodgers A. Polypill: Progress and challenges to global use—Update on the trials and policy implementation. Current Cardiology Reports. 2015; 17(12): 121. DOI: 10.1007/s11886-015-0673-x 26497041

[18] Webster R, Murphy A, Bygrave H, Ansbro É, Grobbee DE, Perel P. Implementing Fixed Dose Combination Medications for the Prevention and Control of Cardiovascular Diseases. Global Heart. 2020 Aug 19; 15(1): 57. DOI: 10.5334/gh.860 32923350 PMC7442173

[19] Peacocke EF, Myhre SL, Foss HS, Gopinathan U. National adaptation and implementation of WHO Model List of Essential Medicines: A qualitative evidence synthesis. PLoS Medicine. 2022; 19(3): e1003944. DOI: 10.1371/journal.pmed.1003944 35275938 PMC8956172

[20] Satheesh G, Dhurjati R, Huffman MD, et al. Standardized treatment protocols for hypertension: global availability, characteristics, and alignment with the hypertension guideline recommendations. Journal of Hypertension. 2024; 42(5): 902–908. DOI: 10.1097/HJH.0000000000003636 38108382

[21] Salam A, Huffman MD, Kanukula R, Prasad EH, Sharma A, Heller DJ, et al. Two-drug fixed-dose combinations of blood pressure-lowering drugs as WHO essential medicines: An overview of efficacy, safety, and cost. Journal of Clinical Hypertension (Greenwich). 2020; 22(10): 1769–1779. DOI: 10.1111/jch.14009 PMC8030031 32815663

[22] Salam A, Praveen D, Patel A, Tewari A, Webster R. Barriers and facilitators to the use of cardiovascular fixed-dose combination medication (Polypills) in Andhra Pradesh, India: A mixed-methods study. Global Heart. 2019; 14(3): 303–310. DOI: 10.1016/j.gheart.2019.07.002 31451238

[23] May CR, Albers B, Bracher M, Finch TL, Gilbert A, Girling M, et al. Translational framework for implementation evaluation and research: A normalisation process theory coding manual for qualitative research and instrument development. Implementation Science. 2022; 17(1): 19. DOI: 10.1186/s13012-022-01191-x 35193611 PMC8861599

