# Correction: A Survey of Availability and Affordability of Polypills for Cardiovascular Disease in Selected Countries 

**DOI:** 10.5334/gh.1350

**Published:** 2024-08-13

**Authors:** Gautam Satheesh, Bishal Gyawali, Marie France Chan Sun, Mark D. Huffman, Amitava Banerjee, Pablo Perel, Adrianna Murphy

**Affiliations:** 1 The George Institute for Global Health, Hyderabad, India; 2 Global Health Section, Department of Public Health, University of Copenhagen, Copenhagen, Denmark; 3 Department of Medicine, University of Mauritius, Mauritius; 4 Washington University in St. Louis, St. Louis, Missouri, US; 5 The George Institute for Global Health, University of New South Wales, Sydney, New South Wales, Australia; 6 Institute of Health Informatics, University College London, London, United Kingdom; 7 Department of Non-Communicable Disease Epidemiology, London School of Hygiene and Tropical Medicine, London, UK; 8 World Heart Federation, Geneva, Switzerland; 9 Department of Health Service Research and Policy, Faculty of Public Health and Policy, London School of Hygiene and Tropical Medicine, London, United Kingdom

**Keywords:** Polypill, Cardiovascular disease, Essential Medicines, Access, Secondary Prevention

## Abstract

This article details a correction to: Satheesh, G., Gyawali, B., Sun, M.F.C., Huffman, M.D., Banerjee, A., Perel, P. and Murphy, A. (2024) ‘A Survey of Availability and Affordability of Polypills for Cardiovascular Disease in Selected Countries’. *Global Heart*. 19(1):p. 56. Available at: https://doi.org/10.5334/gh.1335.

## Correction

The initially published version of the article indicated that “The concept of combining antihypertensives with statins and aspirin for the secondary prevention of CVD was first introduced by the World Health Organization (WHO) in 2001”. In fact, the concept was proposed in 2000 in patents GB 0100548.7 and GB 008791.6 filed by Malcolm Law and Nicholas Wald and discussed in 2001 by a group of experts from WHO and the Wellcome Trust.

## References

[1] Satheesh G, Gyawali B, Sun MFC, Huffman MD, Banerjee A, Perel P, Murphy A. ‘A Survey of Availability and Affordability of Polypills for Cardiovascular Disease in Selected Countries’. Global Heart. 2024; 19(1):p. 56. doi:10.5334/gh.1335 38973984 PMC11225556

[2] Wald NJ, Law MR. Formulation for the Prevention of Cardiovascular Disease. UK Patents GB008791 and GB0100548; 2000.

[3] World Health Organization. Secondary prevention of non-communicable disease in low and middle income countries through community-based and health service interventions. Geneva. 2002; 1–3 August.

