# Supplementary material for: A Survey of Availability and Affordability of Polypills for Cardiovascular Disease in Selected Countries
Source: Glob Heart. 2024 Jul 1;19(1):56. doi: 10.5334/gh.1335 (PMC11225556; doi:10.5334/gh.1335)
Supplement: Supplementary file. — Survey data collection tool. [file gh-19-1-1335-s1.pdf]

Health system factors and access to the polypill for secondary prevention of CVD

Definition: *For the purposes of this project, "polypill refers to a fixed-dose combination of two or more antihypertensive medications and a statin, with or without aspirin in one pill."*

1. Your country of residence

____

2. Name of the city/town/district of residence

____

3. Would you describe this city/town/village as urban, semi-urban or rural? (choose one)

A. Urban (municipality/sub-metrpolitan/metropolitan area)

B. Rural (villages council/gaupalika)

3. Which of these best describes you?

A. General practitioner
B. Cardiologist
C. Pharmacist
D. Epidemiologist
E. Other: ___________________________________

4. Is any polypill currently authorized for marketing in your country?

A. Yes (Go to Q5)
B. No (Go to Q6)

5. If the answer is yes, please provide the following details of the authorised polypills.

Note*: In the first column, list the generic names of each component of the polypill (e.g., lisinopril + hydrochlorothiazide + rosuvastatin + aspirin). In the second column, list the brand name followed by the manufacturing company name in brackets; for example, Zestoretic (AstraZeneca). In the third column, list the strength of each component in milligrams, e.g., 20 + 12.5 + 10 + 75. In the 4th or 5th or 6th columns, provide the names of the guidelines (e.g:), or essential medicine lists, or other lists that include the given polypill, including the source of information or URL.*

**Polypill combination**	**Brand name (manufacturer name)**	**Strength (mg)**	**Name of national/local guideline** (provide source)	**Name of essential medicine list(s)** (provide source)	**Name of other guidelines/ lists/ formularies** (provide source)

6. Does your country locally produce/manufacture one or more polypill combinations for secondary prevention of CVD polypill combinations? If yes, mention the names of the manufacturers.

   ***Note**: Do not consider those companies in your country that simply act as importers/distributors.*

   A. Yes
   B. No
   C. Not sure

7. Additional Notes for Q.1–Q.7:

   ____

   ____

   ____

____

National Health Sector Policy

8. Are polypills covered by any health insurance schemes in your country? If yes, mention the name(s) of the national/social scheme.

   A. Yes _______________________________________
   B. No

9. Are polypills included in the training curriculum for health workers (primary and secondary care)?
   Note*: If you are unsure of this response, please try to get the information or confirm the response by checking the curriculum of the medical and allied health programmes where you live, or checking with an expert.*

   A. Yes
   B. No

10. Additional Notes for Q.8 and Q.9:

____
____
____
____

Health Service Delivery

Instructions

i. *Please select at least 3–5 (ideally 8–10) public/government health facilities (primary, secondary and tertiary) that dispense medications and 3 private pharmacies.*

ii. *These facilities should be from within one city/town/district, not be distributed across a province/country.*

iii. *Where possible, select metropolitan area(s) for the survey; medicines' availability is usually higher in those regions and would reflect "best-case" scenario for the country.*

iv. *Where feasible, consider repeating the above (points i.–iii.) in >1 metropolitan area, town or district.*

11. Please indicate whether the polypills authorized in your country are available in the following:

Note: For each facility type, mention the number of facilities wherein a given polypill was available / total number of surveyed facilities; for e.g., 2/3.

Name of the Polypill	Availability in the public sector (government) hospital pharmacies or dispensaries?	Availability in private retail pharmacies	Availability in government subsidized or semi-public discount pharmacies

12. What is the median unit price (price/tab) of the available polypill(s)?

Note: Please provide the price per tablet or capsule. By price, we refer to the price that a patient would have to pay to purchase the medicine, not the price paid by the pharmacy or the health system. It is possible that the pharmacist may provide you the price of the pack; ensure you collect the number of tablets in each pack to calculate price per tablet/capsule. Finally, report the median unit price (of all the prices collected from various facilities) for each specific polypill. If a given polypill is free in all public sector pharmacies, please enter 0 as the median price.

Name of the polypill	Median unit price (price/tab) in private retail pharmacies	Median unit price (price/tab) in subsidized/special pharmacies

13. Please state the lowest daily wage of a paid worker in your area? Please provide the source of this information (with URL if available).

    ____

14. In your opinion, what might be the top three factors affecting the access to the polypill where you live?

1. ______________________________
2. _______________________________
3. _____________________________
